# Circulating Vitamin D Level and Its Impact on Mortality and Recurrence in Stage III Colorectal Cancer Patients: A Systematic Review and Meta-Analysis

**DOI:** 10.3390/cancers15113012

**Published:** 2023-05-31

**Authors:** Alessandro Ottaiano, Sergio Facchini, Mariachiara Santorsola, Guglielmo Nasti, Gaetano Facchini, Liliana Montella, Nicola Maurea, Marco Cascella, Domenico Iervolino, Bianca Arianna Facchini, Monica Montopoli, Pierluigi Consolo, Vincenzo Quagliariello, Luca Rinaldi, Massimiliano Berretta

**Affiliations:** 1 Istituto Nazionale Tumori di Napoli, IRCCS “G. Pascale”, 80131 Naples, Italy; a.ottaiano@istitutotumori.na.it (A.O.); mariachiara.santorsola@istitutotumori.na.it (M.S.); g.nasti@istitutotumori.it (G.N.); m.cascella@istitutotumori.na.it (M.C.); domenico.iervolino608@gmail.com (D.I.); 2 Division of Medical Oncology, Department of Precision Medicine, University of Campania Luigi Vanvitelli, 80138 Naples, Italy; sergio.facchini@unicampania.it (S.F.); biancaarianna.facchini@studenti.unicampania.it (B.A.F.); 3 Oncology Complex Unit, “S. Maria delle Grazie” Hospital, ASL NA2 NORD, 80078 Pozzuoli, Italy; gaetano.facchini@aslnapoli2nord.it (G.F.); liliana.montella@aslnapoli2nord.it (L.M.); 4 Division of Cardiology, IRCCS “G. Pascale”, 80131 Naples, Italy; n.maurea@istitutotumori.na.it (N.M.); v.quagliariello@istitutotumori.na.it (V.Q.); 5 Department of Pharmaceutical and Pharmacological Sciences, University of Padova, 35122 Padova, Italy; monica.montopoli@unipd.it; 6 Unit of Digestive Endoscopy, University of Messina, Hospital “G. Martino”, 98121 Messina, Italy; pconsolo@unime.it; 7 Department of Advanced Medical and Surgical Sciences, University of Campania “Luigi Vanvitelli”, 80138 Naples, Italy; lucarinaldi@hotmail.it; 8 Department of Clinical and Experimental Medicine, University of Messina, 98122 Messina, Italy

**Keywords:** colorectal cancer, prognosis, vitamin D, survival, disease-free survival

## Abstract

**Simple Summary:**

We conducted a systematic review and meta-analysis to investigate the relationship between pre-operative vitamin D (VD) levels and time-to-outcome in stage III colorectal cancer (CRC) patients. Four articles were included in the analysis, with pooled data from 2628 patients for overall survival and 2024 patients for disease-free survival. The results showed that patients with lower levels of VD had a 38% and 13% increased risk of death and recurrence, respectively, according to random-effects models. These findings suggest that a low VD concentration negatively impacts the time-to-outcome in stage III CRC.

**Abstract:**

Background: Vitamin D (VD) has been implicated in several diseases, including colorectal cancer (CRC). This study aimed to determine whether there is an association between VD levels and time-to-outcome in stage III CRC patients through a systematic review and meta-analysis. Methods: The study adhered to the PRISMA 2020 statement. Articles were searched in PubMed/MEDLINE and Scopus/ELSEVIER. Four articles were selected, with the primary objective of providing a pooled estimate of the risk of death specifically in stage III CRC patients based on pre-operative VD levels. Study heterogeneity and publication bias were analyzed using Tau^2^ statistics and funnel plots. Results: The selected studies showed significant heterogeneity regarding time-to-outcome, technical assessments, and serum VD concentration measures. The pooled analysis of 2628 and 2024 patients revealed a 38% and 13% increase in the risk of death (HR: 1.38, 95% CI: 0.71–2.71) and recurrence (HR: 1.13; 95% CI: 0.84–1.53), respectively, for random-effects models among patients with lower levels of VD. Conclusions: Our findings suggest that a low concentration of VD has a significant negative impact on time-to-outcome in stage III CRC.

## 1. Introduction

Colorectal cancer (CRC) is a neoplasm commonly diagnosed worldwide, ranking third in incidence (1,931,590 new cases per year) and second in mortality (935,180 deaths per year) [1]. Roughly 70% of CRC cases are diagnosed as non-metastatic at stages I–III [2]. Stage I disease typically requires clinical follow-up only, while the use of adjuvant chemotherapy for stage II colon cancer patients remains controversial. According to the current guidelines, chemotherapy should be reserved for high-risk patients with biologic features, such as T4, inadequate lymph node sampling, bowel obstruction, MSS, etc. [3,4]. Treatment decisions in these cases should consider various clinical, personal, and molecular factors, and be based on individualized risk assessments. However, the benefits of adjuvant chemotherapy for this patient population still remain uncertain. On the other hand, adjuvant chemotherapy should be administered to stage III colon cancer patients with loco-regional lymph nodes involved. This therapeutic approach, involving the use of fluoropyrimidines and oxaliplatin after radical surgical removal of the primary tumor, has been shown to be effective in achieving a cure, reducing the risk of death by 20–30% compared to placebo or observation [5,6,7].

Despite this success, CRC can still progress locally and/or distantly, particularly to the liver, thereby reducing the chances of a cure and the patient’s prognosis. Therefore, it is crucial to identify patients within this group who would benefit most from different therapeutic approaches or monitoring strategies. Several innovative biomarkers have been associated with the risk of relapse and death after radical surgery, including cancer gene signatures, protein expression, and soluble molecules [8,9,10]. For example, the concomitant overexpression of CXCR4 and VEGF has been strongly associated with early relapse in stage II–III CRC [11]. However, their use in clinical practice is limited by heterogeneous expression and technical issues, and none of these biomarkers have been adopted for routine use.

Among the soluble molecules, vitamin D3 (VD) is a fat-soluble substance that originates from 7-dehydrocholesterol and is subsequently activated by ultraviolet light [12]. After entering the bloodstream, VD is converted to 25-hydroxyvitamin D (25(OH)D), also known as calcidiol, by the enzyme 25-hydroxylase in the liver. This is the major circulating form of vitamin D and is commonly used to determine individual levels. Subsequently, 25(OH)D is transported to the kidneys, where it undergoes a second hydroxylation step by the enzyme 1α-hydroxylase to form 1,25(OH)2D, also known as calcitriol. Calcitriol binds to different cytosolic receptors and elicits its effects by translocating to the nucleus, essentially modulating gene expression [13]. This reprogramming leads to the regulation of calcium homeostasis. Despite its critical role in calcium absorption and bone resorption [14], VD is also involved in other clinical entities, such as cancer and cardiovascular diseases [15,16].

Previous meta-analyses have reported a favorable impact of VD on cancer-specific survival in colorectal cancer (CRC). However, some studies exploring CRC susceptibility [17] included patients who received external VD supplementation [18,19], or included patients with all stages of the disease without differentiating the prognostic effect of low vs. high VD levels in non-metastatic, stage III CRC patients [20,21].

In this systematic review and meta-analysis, we assessed whether VD levels are associated with time-to-outcome in stage III CRC patients, which is the typical setting of adjuvant systemic interventions. We provide a pooled and updated estimate of the risk of death and progression in stage III CRC patients according to VD levels.

## 2. Methods

This manuscript presents a systematic review and meta-analysis that investigates the association between the circulating level of VD (intended as 25(OH)D) at the time of diagnosis and survival outcomes in patients with stage III CRC. The study was conducted in accordance with the PRISMA statement 2020, and a structured protocol registered in PROSPERO (CRD42023401378) was established at the outset. The selection criteria and methods were explicitly defined in this protocol.

### 2.1. Selection Criteria

**Figure 1:**
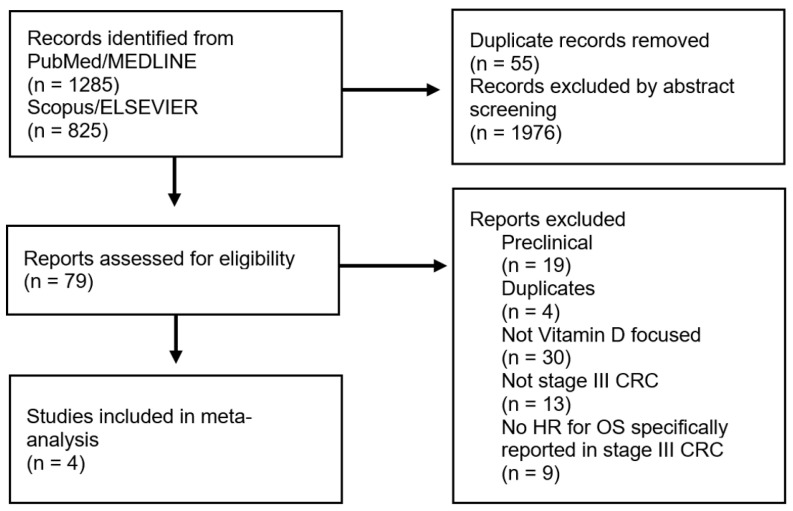
PRISMA flowchart reporting the criteria for the study selection (**left side**) and exclusion (**right side**).

To identify the studies, three independent researchers searched two major international paper databases, PubMed/MEDLINE and Scopus/ELSEVIER, using the following keywords: “colorectal cancer” OR “colon” OR “rectal” AND “vitamin D” OR “cholecalciferol” OR “calcidiol”. Articles published between 2002 and 2022 were searched (accessed on 31 July 2022). The selection criteria for study enrollment were: 1. articles written in English to avoid language and publishing biases, 2. patients aged 18 years and older, 3. histologically diagnosed with stage III CRC, 4. VD dosed at the time of diagnosis, before surgery (post-operative cohorts were excluded from the analysis), and 5. an explicit indication of the hazard ratio (HR) (according to VD levels) for overall survival (including 95% confidence intervals, 95% CIs) specifically calculated for stage III CRC patients. The information about HR was sought anywhere in the selected articles. There were no limitations on VD supplementation, study design, or possible adjuvant therapy administered. Preclinical articles that focused on in vitro and/or animal experiments were excluded. Articles that evaluated VD and other biomarkers were included only if VD was independently studied. The complete flowchart for the selection process is presented in Figure 1.

### 2.2. Data Extraction

For each study, we extracted the following information: the first author, year of publication, study design, clinical–pathological characteristics of patients, methodology for assessing VD levels, number of enrolled patients, follow-up time, time-to-outcome including HRs and 95% CIs. To minimize the risk of confounding effects, we extracted the maximally adjusted HR from each paper. Three investigators (A.O., S.F., M.B.) independently reviewed all data, and any criticisms or discrepancies were discussed with all authors.

### 2.3. Principal Endpoint

The primary objective of this meta-analysis was to estimate the pooled risk of death in patients with stage III CRC based on their circulating VD levels before surgery.

### 2.4. Quality Assessment

To ensure a formal assessment of the quality and risk of bias, four authors (A.O., S.F., M.S., and M.B.) were responsible for managing the rating of the methodologies and results of the selected studies. We used the Methodological Index for Non-Randomized Studies (MINORS) criteria [22] and the Newcastle–Ottawa Scale [23] for evaluation. The final scores were independently rated by D.I. and M.C. with blinding to previous results. Any discrepancies were resolved through consensus discussions involving all authors.

### 2.5. Statistical Methods

A meta-analysis was conducted to examine the correlation between VD levels and the risk of death in stage III CRC patients. Disease-free survival (DFS) was also examined as a secondary outcome. A random-effects model was utilized due to the heterogeneity observed among the studies, utilizing the Hartung–Knapp–Sidik–Jonkman approach [24]. Under this model, true effects are believed to differ between the studies, and the summary effect is the weighted average of the effects reported in different studies. This method produces a more cautious estimate of the pooled HR and is favored in the presence of heterogeneity. Results were presented using Forest plots, displaying HRs and 95% CIs, as well as a final pooled HR. An HR of 1.0 indicates that the event probability (EP) is the same in both high- and low-VD-level groups (EP VD low/EP VD high). However, if the HR is greater than 1.0, it implies that the low VD level group is at a higher risk of death or progression. In situations where the HR reports high VD levels in the numerator (VD high vs. low), the HRs and CIs were recalculated based on Altman et al. [25] to ensure that the comparison trajectory is consistent between VD low vs. VD high (calculated HR VD low vs. high was 1/HR VD high vs. low), making it easier for readers to comprehend. The degree of variation among the studies (heterogeneity) was assessed using I^2^ and Tau^2^ statistics. I^2^ calculates the proportion of observed variation that is due to genuine differences rather than chance. I^2^ = 100% × (Q − DF)/Q, where Q represents Cochran’s heterogeneity statistic and DF represents the degrees of freedom. Negative I^2^ values are set to zero to ensure that I^2^ falls between 0% and 100%. A value of 0% suggests no observed heterogeneity, while larger values indicate increasing heterogeneity. In addition to I^2^, Tau^2^, which is a measure of the between-study variance that takes into account the size of the individual studies, was also used to assess the degree of heterogeneity among the studies in this meta-analysis. Unlike I^2^, which provides a relative measure of heterogeneity, Tau^2^ estimates the absolute magnitude of the between-study variance. The formula for Tau^2^ is Tau^2^ = (Q − DF)/[(k − 1) × sum of inverse variances], where k is the number of studies included in the meta-analysis. Tau^2^ is especially useful when the studies have varying sample sizes and effect estimates [26]. Potential publication bias was evaluated graphically using a funnel plot [27]. The statistical analyses were conducted using the MedCalc Statistical Software (MedCalc^®®^ Statistical Software version 19.6, MedCalc Software Ltd., Ostend, Belgium) and R studio software version 4.1.1 (R studio Inc. Company, Boston, MA, USA).

## 3. Results

Four studies were included in this meta-analysis [28,29,30,31]. Figure 1 shows the selection flowchart.

**Figure 2:**
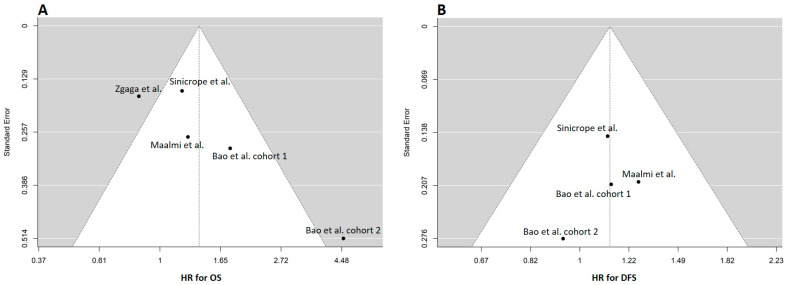
Funnel plots of selected studies for overall survival (OS) (**A**) and disease-free survival (DFS) [28,29,30,31]. (**B**) The estimate of the outcome is reported in the x-axis, while the y-axis typically represents the standard error [28,29,30]. The funnel plot in OS (**A**) shows two studies that are not symmetrically distributed (this asymmetry suggests the possibility of publication bias). In (**B**), which depicts PFS, there is no observed asymmetry in the funnel plot. The studies appear to be distributed symmetrically around the overall estimate, indicating that publication bias or small-study effects may not be present in this particular analysis.

The studies selected for analysis demonstrated substantial heterogeneity in terms of OS, as indicated by the Tau^2^ values (Tau^2^: 0.16; Cis: 0.012–3.2). Funnel plots for OS in two of the studies (the second cohort of Bao et al. [29] and Zgaga et al. [31]) revealed clear asymmetries, suggesting a significant publication bias in these articles (Figure 2A). Conversely, no clear asymmetries were observed in the funnel plots for DFS (Figure 2B).

Table 1 reports the technical and methodological characteristics of the studies. Three out of the four studies were retrospective analyses. All studies met the principal characteristic of presenting an independent HR analysis for stage III CRC patients. Technical assessments of serum VD level determination were heterogeneous. VD concentration was adjusted per season in two studies. Information about eventual VD supplementation was reported only in one study. Furthermore, VD concentration was expressed through different measurement units. Two articles expressed concentration in nmol/L, while two expressed it in ng/mL. Concentration cutoffs (low vs. high) were also heterogeneous. When expressed in nmol/L, the cutoff definition for the low level ranged from 45.2 to 75 nmol/L. A similar variability was observed in the ng/mL subgroup (range 13.25–30 ng/mL). The enrolled articles’ quality scores were equal to or greater than 5 for the MINORS and NOS scales, respectively.

Table 2 reports the clinical–pathological characteristics of patients included in the selected articles. The median age ranged from 62.5 to 69 years and was similar across all papers. The cutoffs for age dichotomization into elderly and non-elderly patients were heterogeneous. The male gender was predominant, and when reported, T3 was the predominant diagnosed T-stage across the studies. The primary tumor side was not specified in two articles.

Table 3 presents the time-to-outcome estimates. The median follow-up was adequate in three studies (>36 months), but was not specified in one study. An adjuvant chemotherapy regimen was administered (as declared in the Patients and Methods sections of the articles), but not detailed in the studies (timing, regimens, toxicities, etc.).

**Figure 3:**
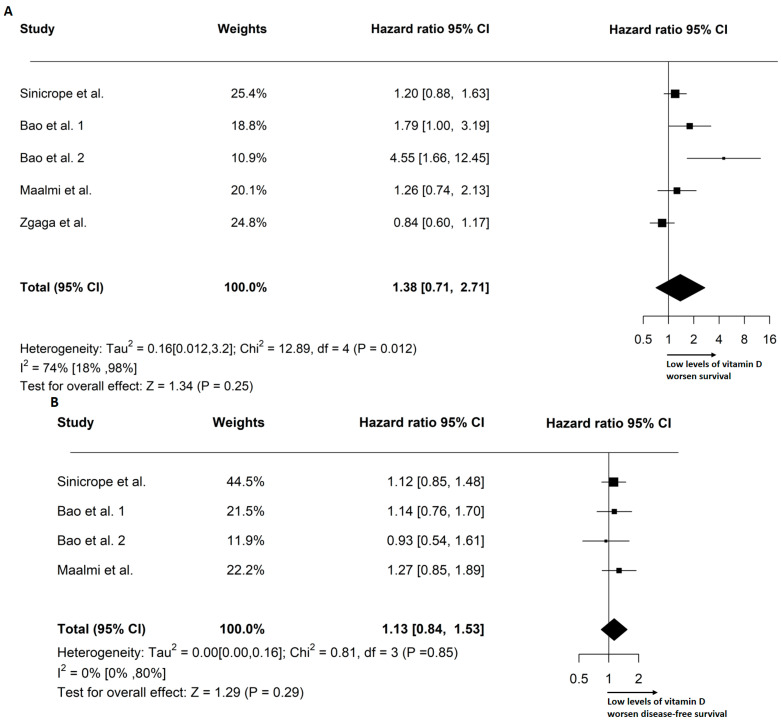
Forest plots depicting the overall survival (**A**) and disease-free survival [28,29,30,31] (**B**) outcomes, stratified by pre-operative circulating levels of VD [28,29,30]. The plots report Tau^2^, I^2^ statistics, and the pooled hazard ratio (HR) based on the random-effects model.

The effect size of the VD levels on prognosis was defined by the HR. Forest plots of the VD effects on OS and DFS are shown in Figure 3. The pooled analysis resulted in a 38% increase in the risk of death in 2628 patients for the random-effects models (HR: 1.38, 95% CI: 0.71–2.71). The pooled progression risk increase in 2024 patients was 13% (HR: 1.13; 95% CI: 0.84–1.53). Based on our findings, a low concentration of circulating VD before surgery has a negative effect on OS and DFS in individuals with stage III CRC.

## 4. Discussion

Although there are some controversial aspects in stage II, adjuvant chemotherapy is currently considered the standard of care for patients with stage III colorectal cancer who have undergone radical surgery. The goal of adjuvant therapy is to eliminate residual cancer cells and achieve definitive cure. Several studies have suggested a positive association between VD levels and cancer outcomes, although results have not been differentiated for stages or the initial tumor burden [32]. In our study, we focused on the effect of VD circulating levels on the prognosis of colorectal cancer stage III patients by selecting studies [28,29,30,31] that reported this effect. Our aim was to investigate the correlation between VD and CRC in terms of influencing the OS and DFS in a clinical context where adjuvant chemotherapy is considered a benchmark, and to provide a more comprehensive and convincing evidence base than what is currently available in fragmented form.

Interestingly, VD supplementation is widely used to treat and prevent VD deficiency, which is prevalent worldwide [33]. The recommended daily intake of VD varies by age and health status, and can be obtained from dietary sources such as fortified foods or supplements [34]. Additionally, adequate sunlight exposure can contribute to VD synthesis in the skin. VD supplements are available in different forms, including cholecalciferol (vitamin D3) and ergocalciferol (vitamin D2), and can be easily administered orally [35]. VD is a fat-soluble vitamin that plays a crucial role in maintaining bone health and calcium homeostasis. Its role in other physiological functions, such as immune response and cellular proliferation, has also been extensively studied [36]. However, only one study included in our meta-analysis provided a description of VD supplementation, mentioning the dietary intake and supplements at different dosage ranges (<5, 5–10, >10 μg/day) [31]. Nevertheless, it was unclear whether the supplementation was regulated or controlled, and the reasons for supplementation were not reported. This information was obtained through the administration of “frequency questionnaires” to patients. Moreover, it was not specified whether the VD intake was self-managed by the patients or prescribed by a doctor, either through food or supplements. These details could shed light on the methodological aspects of the selected articles. Regrettably, controlled and prospective studies assessing the potential benefits of VD supplements (type, dosage, timing, etc.) on the time-to-outcome, achievable with adjuvant therapies for stage III CRC, are entirely lacking. Therefore, a direct piece of evidence indicating the importance of maintaining high levels of VD to prevent cancer recurrence in CRC patients is also absent.

Recently, a growing body of literature has focused on the association between VD and cancer [37]. Its active form, 1,25-dihydroxyvitamin D, binds to the VD receptor (VDR) in various tissues, including the bone, intestine, and immune cells. VDR activation regulates calcium and phosphate metabolism, which is essential for skeletal health. Additionally, it influences cell differentiation and proliferation, and has anti-inflammatory and anti-carcinogenic properties [38]. In recent years, research has suggested that VD has a protective effect against several types of cancer. Mechanistically, VD exerts an anti-proliferative effect on cancer cells by inducing cell cycle arrest and apoptosis, and reducing angiogenesis and metastasis [39]. Moreover, VD modulates the immune response, leading to the activation of natural killer cells and the inhibition of pro-inflammatory cytokines [40], which can have positive impacts on chemotherapy efficacy and toxicity [41,42].

Furthermore, VD has garnered considerable attention for its potential role in potentiating and/or synergizing the efficacy of chemotherapy in the context of CRC treatment. Both in vivo and in vitro studies have provided insights into the anti-tumor effects observed when combining VD with anti-cancer treatments. In vitro investigations have demonstrated that VD can enhance the cytotoxic effects of chemotherapy agents, leading to increased cancer cell death [43]. It has been suggested that it can modulate molecular pathways involved in apoptosis, cell cycle regulation, thereby sensitizing cancer cells to chemotherapy-induced cytotoxicity. In vivo studies using animal models of CRC have provided additional evidence supporting the potential benefits of combining VD with chemotherapy. These studies have shown that VD supplementation can enhance the anti-tumor activity of chemotherapeutic agents, leading to reduced tumor growth, improved survival rates, and decreased metastasis [44,45]. While the exact mechanisms underlying the synergistic effects of VD and chemotherapy remain to be fully elucidated, the accumulating evidence suggests a promising therapeutic strategy for CRC treatment [46,47]. However, future clinical trials are needed to validate these findings and determine the optimal dosing, timing, and patient selection for this combined approach.

Our meta-analysis reveals an increased risk of death and progression in stage III patients with low circulating levels of VD before surgery, suggesting potential beneficial effects of VD supplementation in this clinical setting. Very interestingly, a recent study suggests that VD can prompt and support immune responses in the early stages of CRC, by modulating T-regulatory-cells (Treg) function [48].

Some limitations of our study need to be highlighted and discussed. First of all, despite our meta-analysis being methodologically sound and rigorous, it is based on a relatively small number of studies, specifically four. This is due to the limited number of studies that have specifically evaluated the prognostic effect of VD in stage III CRC patients. It is worth noting, however, that this represents an exceptionally interesting clinical setting as it pertains to the treatments (adjuvant chemotherapy) associated with patient recovery. In this regard, we hope that the scientific community will generate further prospective findings regarding the role of VD levels in this clinical scenario. Although the included studies had large sample sizes, long follow-up times, and good quality scores according to the MINORS and NOS scales, they demonstrated heterogeneity in methodological issues, such as VD assessment methods and cutoffs used to distinguish between low and high VD levels. Additionally, all but one study were retrospective. Lastly, the selected studies reported that stage III patients received adjuvant chemotherapy based on standard guidelines, but they did not specify the number of patients who were actually treated or the type of adjuvant therapies administered. Together, these factors may introduce unknown biases that could negatively impact the reliability of pooled data, and they should be taken into account when interpreting our results.

## 5. Conclusions

It has been well-established that VD possesses anti-carcinogenic properties and modulates the immune response, which can enhance the effectiveness of chemotherapy. Based on the results of our meta-analysis, future studies should investigate whether VD levels can serve as an additional prognostic stratification factor, and if supplementation in this clinical setting could be an innovative approach to improving clinical outcomes.

## Figures and Tables

**Table 1:** Study characteristics and quality.

First Author, Year	Study Design	Vitamin D Assessment	Vitamin D Adjusted per Season	Vitamin D Supplementation?	No. of AJCC Stage III Patients	TTO	Comparison Modalities	Other Biomarkers Evaluated	MINORS Scores	NOS Score
Sinicrope, 2021 [28]	R	RIA	No	NS	600	OS, DFS	Insufficient vs. Sufficient (cutoff: 30 ng/mL)	Adiponectin	5	5
Bao, 2020 [29]	R	EBA	No	NS	484	OS, DFS	High vs. Low (cutoff: 75 nmol/L)	No	8	7
Maalmi, 2017 [30]	R	HPLC-ESIMS	Yes	NS	940	OS, DFS	Low vs. High (interquintile comparison Q1–4 vs. Q5, cutoff: 45.2 nmol/L)	No	8	7
Zgaga, 2014 [31]	P	LC/MS	Yes	Yes	604	OS	Low vs. High (intertertile comparison, T1, T2 vs. T3, cutoff: 13.25 ng/mL)	Genotype at the VDR locus	7	7

DFS: disease-free survival; EBA electrochemiluminescence binding assay; h: haplotype; HPLC-ESIMS: high-performance liquid chromatography-electro spray ionization mass spectrometry; LC/MS: liquid chromatography-tandem mass spectrometry; MINORS: Methodological Index for Non-Randomized Studies; NOS: Newcastle–Ottawa Scale; OS: overall survival; P: prospective; R: retrospective; RIA: radioimmunoassay; TTO: time-to-outcome; VDR: vitamin D receptor.

**Table 2:** Clinical–pathological characteristics of entire patients cohorts included in the selected articles.

Author	Year	Age			Gender		T		Side	
		Young	Old	Median	Male	Female	≤2	≥3	Left	Right
Sinicrope et al. [28]	2021	≤65: 420	>65: 180	NR	316	284	96	504	286	303
Bao et al. [29]	2020	<60: 325	≥60: 304	61	424	304	40	688	490	237
Maalmi et al. [30]	2017	<70 *: 1524	≥70: 1386	69	1732	1178	NR	NR	NR	NR
Zgaga et al. [31]	2014	NR	NR	62.56	917	681	NR	NR	NR	NR

* The authors consider 65 years as the cutoff discerning young vs. old; however, in the text, the categorical classes reported are (30–59; 60–69; 70–79; >80); NR: not reported.

**Table 3:** Detailed risk of death and progression in the selected articles.

Author	Year	Median Follow-Up (months)	Median OS (months)		* HR_OS_	CI_OS_	*P*_OS_	Median DFS (months)	* HR_DFS_	CI_DFS_	*P*_DFS_
Sinicrope et al. [28]	2021	NR	NR		1.20	0.88–1.63	0.25	NR	1.12	0.84–1.47	0.43
Bao et al. [29]	2020	53 (in both cohorts)	NR	Cohort 1	0.56	0.31–0.99	0.047	NR	0.88	0.59–1.32	0.884
				Cohort 2	0.22	0.08–0.60	0.003	NR	1.07	0.62–1.83	0.791
Maalmi et al. [30]	2017	57	NR		1.26	0.74–2.12	0.67	NR	1.27	0.86–1.90	0.80
Zgaga et al. [31]	2014	106.8	32.4		0.84	0.60–1.17	0.30	NR	NR	NR	NR

* If necessary, HR was transformed in a forest plot (see Section 2) to harmonize the comparison trajectory. CI: Confidence interval; DFS: disease-free survival; NR: not reported; OS: overall survival; P: *p*-value.

## Data Availability

The R script for conducting the meta-analysis is available at https://zenodo.org/record/7907127#.ZFjEFHZBxD8 last accessed on 19 April 2023.
